# Molecular breeding for the development of multiple disease resistance in Basmati rice

**DOI:** 10.1093/aobpla/pls029

**Published:** 2012-11-02

**Authors:** Atul Singh, Vikas K. Singh, S. P. Singh, R. T. P. Pandian, Ranjith K. Ellur, Devinder Singh, Prolay K. Bhowmick, S. Gopala Krishnan, M. Nagarajan, K. K. Vinod, U. D. Singh, K. V. Prabhu, T. R. Sharma, T. Mohapatra, A. K. Singh

**Affiliations:** 1 Division of Genetics, Rice Section, Indian Agricultural Research Institute, New Delhi 110 012, India; 2 Department of Genetics and Plant Breeding, C.C.S. University, Meerut 200 005, India; 3 Division of Plant Pathology, Indian Agricultural Research Institute, New Delhi 110 012, India; 4 Rice Breeding and Genetics Research Centre, Aduthurai 612101, Tamil Nadu, India; 5 National Research Centre on Plant Biotechnology, Indian Agricultural Research Institute, New Delhi 110 012, India; 6 Current address: Central Rice Research Institute, Cuttack 753 006, Odisha, India

## Abstract

**Background and aims:**

Basmati rice grown in the Indian subcontinent is highly valued for its unique culinary qualities. Production is, however, often constrained by diseases such as bacterial blight (BB), blast and sheath blight (ShB). The present study developed Basmati rice with inbuilt resistance to BB, blast and ShB using molecular marker-assisted selection.

**Methodology:**

The rice cultivar ‘Improved Pusa Basmati 1’ (carrying the BB resistance genes *xa13* and *Xa21*) was used as the recurrent parent and cultivar ‘Tetep’ (carrying the blast resistance gene *Pi54* and ShB resistance quality trait loci (QTL), *qSBR11-1*) was the donor. Marker-assisted foreground selection was employed to identify plants possessing resistance alleles in the segregating generations along with stringent phenotypic selection for faster recovery of the recurrent parent genome (RPG) and phenome (RPP). Background analysis with molecular markers was used to estimate the recovery of RPG in improved lines.

**Principal results:**

Foreground selection coupled with stringent phenotypic selection identified plants homozygous for *xa13, Xa21* and *Pi54*, which were advanced to BC_2_F_5_ through pedigree selection. Marker-assisted selection for *qSBR11-1* in BC_2_F_5_ using flanking markers identified seven homozygous families. Background analysis revealed that RPG recovery was up to 89.5%. Screening with highly virulent isolates of BB, blast and ShB showed that the improved lines were resistant to all three diseases and were on a par with ‘Improved Pusa Basmati 1’ for yield, duration and Basmati grain quality.

**Conclusions:**

This is the first report of marker-assisted transfer of genes conferring resistance to three different diseases in rice wherein genes *xa13* and *Xa21* for BB resistance, *Pi54* for blast resistance, and a major QTL *qSBR11-1* have been combined through marker-assisted backcross breeding. In addition to offering the potential for release as cultivars, the pyramided lines will serve as useful donors of gene(s) for BB, blast and ShB in future Basmati rice breeding programmes.

## Abstract (precis)

Marker assisted backcross breeding for combining three resistance genes (*xa13* and *Xa21* for Bacterial Blight, *Pi54* for blast) and a major QTL (*qSBR11-1* for resistance to Sheath blight) in Basmati rice.

## Introduction

Basmati rice cultivated in the north-western part of the Indo-Gangetic plains of the Indian subcontinent is highly valued in the international market due to its unique combination of aroma, grain, cooking and eating qualities (Singh *et al.* 1988, 2000). Basmati rice worth $2 billion was exported from India during 2009–10 (Website 1). Basmati rice germplasm including improved cultivars are highly susceptible to biotic and abiotic stresses. Among the biotic stresses, bacterial blight (BB) caused by *Xanthomonas oryzae* pv. *oryzae*, blast caused by *Magnoporthae oryzae* and sheath blight (ShB) caused by *Rhizoctonia solani* not only cause severe yield losses but also impair the quality of the rice grain (Singh *et al.* 2011).

Globally, rice blast causes annual yield losses of up to 50 % (Scardaci *et al.* 1997). Sheath blight also causes major crop loss worldwide (Ou 1985), and in India yield loss of up to 54.3 % has been reported (Chahal *et al.* 2003). In the case of Basmati rice, economic losses from reduced yield are amplified by a severe deterioration in quality. Although these diseases can be managed by fungicides, breeding resistant varieties with durable resistance is a more ecologically sound and sustainable approach.

A large number of genes conferring resistance have been reported. These include a major gene *Pi54*. This encodes a protein containing a nucleotide-binding leucine-rich repeat domain that provides resistance to the predominant races of the pathogen in India (Sharma *et al.* 2005). Resistance to the ShB pathogen is quantitative in nature (Pinson *et al.* 2005) and 16 quality trait loci (QTLs) providing ShB resistance across different genetic backgrounds have been identified in different QTL mapping studies (Srinivasachary *et al.* 2011). *qSBR11-1* is a major QTL that has been found to be effective against the ShB pathogen consistently over time and at different locations (Channamallikarjuna *et al.* 2010). ‘Tetep’, an *indica* rice cultivar from Vietnam, is the source of resistance to both blast (*Pi54*) and ShB (*qSBR11-1*) diseases. In addition, ‘Tetep’ is an invaluable donor of several other resistance genes, including *Xa1*, *Xa2*, *Xa12* and *Xa16* for BB resistance (Nelson *et al*. 1994; Goel *et al*. 1998); *Pita* (Jia *et al*. 2003), *Pi1(t), Pi4^a^(t), Pi4^b^(t), Pi3(t)* (Inukai *et al*. 1994)*, Pi-k^h^* (Xu *et al.* 2008) and *Pi54* (Rai *et al.* 2011) for blast resistance; and 10 other QTLs for ShB resistance (Channamallikarjuna *et al*. 2010).

The long history of rice cultivation and favourable climatic factors in tropical Asia are conducive to many diseases (including BB, blast and ShB) that pose a constant threat to rice production (Khush 1989). Consequently, breeding cultivars with multiple disease resistance would help sustain rice productivity. Marker-assisted selection (MAS) is an approach that has enabled efficient and precise transfer of genes/QTL(s) in many crop species, and offers a fast and efficient alternative to conventional breeding and selection methods. Marker-assisted selection has been shown to be highly efficient for the precise transfer of major genes for BB resistance (Huang *et al*. 1997; Joseph *et al*. 2004; Gopalakrishnan *et al*. 2008; Basavaraj *et al*. 2010), blast resistance (Singh *et al*. 2012) and QTLs for ShB resistance (Tan *et al.* 2005; Pinson *et al*. 2008; Zuo *et al.* 2008; Wang *et al.* 2012) in rice.

Marker-assisted backcross breeding (MABB) coupled with phenotypic selection for agronomic, grain and cooking quality traits has been used to incorporate BB resistance genes *xa13* and *Xa21* into ‘Pusa Basmati 1’ (Joseph *et al.* 2004). One of the improved lines was released as ‘Improved Pusa Basmati 1’ for commercial cultivation in 2007 (Gopalakrishnan *et al.* 2008), and this is one of the first products of MAS to be used in India. However, the susceptibility of ‘Improved Pusa Basmati 1’ and other Basmati rice varieties to rice blast and ShB disease remains a major concern. These problems are further accentuated by the non-availability of resistant sources for these diseases in the Basmati germplasm.

In the current study, the cultivar ‘Tetep’ was used as donor mainly for the transfer of the blast resistance gene, *Pi54*, through marker-assisted foreground selection in combination with stringent phenotypic selection for recovery of agro-morphological, grain and cooking quality traits of the recurrent parent ‘Improved Pusa Basmati 1’. However, ‘Tetep’, the donor for the blast resistance gene, *Pi54*, also possesses a major QTL for ShB resistance, *qSBR11-1*, on the arm of chromosome 11, in which *Pi54* is located. Therefore, the superior advanced lines possessing both BB- and blast-resistant genes were also analysed for the possible introgression of this major QTL for ShB resistance through MAS. Here we report the use of MAS for combining resistance to three diseases, namely BB, blast and ShB, in Basmati rice.

## Materials and methods

### Plant materials and development of improved lines

The Basmati rice cultivar ‘Improved Pusa Basmati 1’, resistant to BB, was used as the recurrent parent, while the cultivar ‘Tetep’ was used as donor for the blast resistance gene, *Pi54*, and ShB resistance QTL, *qSBR11-1*. A single F_1_ plant was backcrossed with ‘Improved Pusa Basmati 1’ to generate the BC_1_F_1_s (the backcross series was designated as ‘Pusa1608’). Marker-assisted foreground selection was employed using gene-linked/gene-based markers for *xa13*, *Xa21* and *Pi54* to identify the plant homozygous for the genes *xa13* and *Xa21*, and heterozygous for the gene *Pi54*. Further, the selected plants were subjected to stringent phenotypic selection for agro-morphological, grain and cooking quality traits. The desirable BC_1_F_1_ plant with maximum recovery of the recurrent parent phenome (RPP) was backcrossed to develop the BC_2_F_1_ generation. The BC_2_F_1_ plants were also subjected to foreground selection followed by phenotypic selection to identify five plants homozygous for *xa13* and *Xa21*, heterozygous for *Pi54* and with maximum recovery for RPP. These plants were then selfed to generate BC_2_F_2_ populations. In the BC_2_F_2_ generation, plants homozygous for all three genes were identified and then advanced to the BC_2_F_5_ generation through the pedigree method of selection.

### DNA extraction and polymerase chain reaction amplification

Total genomic DNA was extracted by the micro-extraction protocol of Prabhu *et al.* (1998). Polymerase chain reaction (PCR) was performed in a thermal cycler (G-Storm, Somerset, UK) using a 10 μL total reaction volume as described previously (Basavaraj *et al.* 2010). This contained 30 ng μL^−1^ of template DNA, 5 pmol of each primer (synthesized from Sigma Inc., St. Louis, MO, USA), 1.5 mM MgCl_2_, 0.2 mM dNTPs (MBI, Fermentas, Vilnius, Lithuania) and 0.5 U of *Taq* polymerase (Bangalore Genei, Bangalore, Karnataka, India). Polymerase chain reaction comprised one cycle of denaturation at 95 °C for 5 min, followed by 35 cycles at 95 °C for 30 s, 55 °C for 30 s and 72 °C for 1 min, with a final extension of 72 °C for 7 min. The amplified products were resolved on 3.5 % Metaphor™ gel (Lonza, Rockland, ME, USA) containing 0.1 µg mL^−1^ of ethidium bromide (Amresco, Solon, OH, USA) along with a DNA size standard ladder (MBI, Fermentas) and documented in a Gel Documentation System (Biorad, Hercules, CA, USA).

### Molecular marker analysis

#### Foreground selection

The gene-based markers for BB resistance genes *xa13* (*xa-13 prom*) and *Xa21* (pTA248), and the gene-linked marker for blast resistance gene *Pi54* (RM206) were used in the foreground selection. The markers RM224, sbq1, sbq11, K39516 and sbq33 (Channamallikarjuna *et al.* 2010) flanking the ShB resistance QTL *qSBR11-1* were analysed for parental polymorphism between ‘Improved Pusa Basmati 1’ and ‘Tetep’, and only one marker, RM224 (27.2 Mb), was found to be polymorphic. Therefore, 10 additional SSR markers from the region flanking (Website 2) *qSBR11-1* were subjected to parental polymorphism, of which the marker RM7443 (physical position—28.3 Mb, same as that of the flanking marker, sbq33) was found to be polymorphic between ‘Tetep’ and ‘Improved Pusa Basmati 1’. The foreground selection for ShB resistance QTL *qSBR11-1* was carried out using the flanking markers RM224 and RM7443 in the segregating generations. Details of the primer sequence, chromosomal location, fragment size, annealing temperature and physical position are presented in Table 1.

**Table 1:** Molecular markers used for foreground selection for BB, blast and ShB resistance genes.

Trait	Gene/QTL	Marker	LG	MD (cM)	Forward sequence	Reverse sequence	Reference
Bacterial blight	*xa13*	xa13-prom	8	0.0	GGCCATGGCTCAGTGTTTAT	GAGCTCCAGCTCTCCAAATG	Singh *et al*. (2011)
	*Xa21*	pTA248	11	0.0	AGACGCGGAAGGGTGGTTCCCGGA	AGACGCGGTAATCGAAGATGAAA	Ronald *et al.* (1992)
Blast	*Pi54*	RM206	11	0.6	CCCATGCGTTTAACTATTC	CGTTCCATCGATCCGTATGG	Sharma *et al.* (2005)
Sheath blight	*qSBR11-1*	*RM224*	11	FM	ATCGATCGATCTTCACGAGG	TGCTATAAAAGGCATTCGGG	Channamallikarjuna *et al.* (2010)
		RM7443	11	FM	ACACTGTACACCACACTTCAGC	CAGGGAAATGACACTGTCCC	

LG, linkage group; MD, map distance; AT, annealing temperature; FM, flanking marker.

#### Background analysis

A set of 435 SSR primer pairs distributed uniformly across the 12 chromosomes of the rice genome was selected for a parental polymorphism survey between the donor ‘Tetep’ and the recurrent parent ‘Improved Pusa Basmati 1’ (Website 2). The SSR markers, which were polymorphic, were then used to identify plants with maximum recovery of recurrent parent genome (RPG) in BC_2_F_5_ generation. Additionally, markers were added in the 5 Mb region on either side of the genes on the respective carrier chromosomes (chromosomes 8 and 11) in order to identify BC_2_F_5_ recombinants with minimum donor segments as well as the target loci. The PCR products were amplified and resolved in 3.5 % Metaphor™ gel. The genomic contribution of the parents in the elite selections was analysed and represented using the software Graphical Geno Types (GGT) Version 2.0 (Van Berloo 1999).

### Screening for BB, blast and ShB resistance

#### Artificial inoculation

A set of 10 plants raised in two replications was screened for BB resistance through artificial inoculation during Kharif (rainy season) 2010 under field conditions at the experimental farm of the Division of Genetics, Indian Agricultural Research Institute (IARI), New Delhi, India. Plants were inoculated with the bacterial suspension at a density of 10^9^ cells/mL at maximum tillering stage using the most virulent isolate ‘Kaul’ of *X. oryzae* pv. *oryzae* (Joseph *et al.* 2004). Five young leaves in each plant were inoculated through the clip inoculation method (Kauffman *et al.* 1973) and the disease reaction was scored 21 days after inoculation (DAI). The weather during Kharif 2010 was conducive for BB infection with the temperature ranging from 21.8 to 31.9 °C and relative humidity in the range 88.0–100 %. Plants with an average lesion length of up to 6 cm were considered resistant and those with lesion lengths >6 cm were scored as susceptible.

Screening for blast resistance used the four most virulent strains (Singh *et al*. 2012) of *M. oryzae* from Basmati-growing regions using standard protocol (Bonman *et al*. 1986). About 30–40 mL of the spore suspension containing gelatin (0.1 %) and Tween-20 (0.02 %) were sprayed onto seedlings using a glass atomizer. Inoculated seedlings were kept in a humid chamber with the temperature maintained at 25 ± 1 °C. Distilled water was sprinkled three to four times a day to maintain high humidity. The disease reaction was recorded 7 DAI using a 0–5 disease scoring scale (Bonman *et al.* 1986). The plants exhibiting reactions that scored 0–3 were considered resistant while those showing reactions that scored 4–5 were categorized as susceptible.

The screening for ShB resistance in the advanced breeding lines was carried out on a set of three hills in two replications in separate plots through artificial inoculation under field conditions at the experimental farm of the Division of Genetics, IARI, New Delhi, India. A virulent isolate of *R. solani* from Kaparthula (*Rs-K*), which was used for mapping QTLs for ShB resistance in ‘Tetep’ by Channamallikarjuna *et al*. (2010), was mass multiplied on shoots of the water sedge, *Typha angustata*. The inoculation was achieved by placing the shoot pieces having mycelium and sclerotia between the tillers in the central region of the rice hills, 5–10 cm above the waterline (Bhaktavatsalam *et al.* 1978). The weather in New Delhi during *Kharif* 2010 (as indicated above) was conducive for the development of ShB disease. The phenotypic observations on vertical spread were recorded 10 and 25 DAI. The relative lesion height (RLH) was calculated using the formula: relative lesion height (RLH) = lesion height/plant height × 100.

The average lesion height over two replications (six lesions) consisting of three tillers in each replication after 25 DAI was used for calculating RLH. Based on the RLH, ShB disease reactions of test entries were grouped into six categories according to Ahn *et al.* (1986). The lines with a disease grade score of 0 were considered as highly resistant, 1 as resistant, 3 as moderately resistant, 5 as moderately susceptible, 7 as susceptible and 9 as highly susceptible.

### Field screening for blast resistance at the Uniform Blast Nursery

All the promising lines were evaluated for their reaction to leaf and neck blast at the Uniform Blast Screening Nursery (UBN) at the Agricultural Research Station (ARS), Mugad, in Karnataka (Southwest India) using standard protocol during Kharif 2010 (Singh *et al*. 2012). A 50-cm-long row of each entry was planted in an upland nursery bed with a row spacing of 10 cm. A row of susceptible check was planted after every five entries and also on the borders to ensure uniform spread of the disease. The weather during Kharif 2010 was conducive for blast infection with a mean temperature of 23.1 °C and a mean relative humidity of 75.1 %, and a maximum humidity of 100 % during the growth period. Data on the blast reaction of the entries were recorded three times using a scale of 0–9 (SES 1996) at 10-day intervals starting 30 days after sowing. The lines with scores of 0–3 were considered resistant, 4–5 as moderately resistant, 6 as moderately susceptible and 7–9 as susceptible.

### Evaluation of agronomic performance and grain quality

The advanced lines along with parental lines were planted at a spacing of 15 × 20 cm in a randomized complete block design with three replications, and were evaluated for agronomic traits during *Khairf* 2010 at the experimental farm of the Division of Genetics, IARI, New Delhi, India. Data on five plants were recorded for various agronomic traits: days to 50 % flowering (DFF), plant height (PH), number of tillers (NT), panicle length (PL), filled grains per panicle (FG/P), spikelet fertility (SF), 1000-grain weight (TW) and yield per plant (Y/P). The analysis of grain quality traits such as grain size, kernel length before cooking (KLBC), kernel length after cooking (KLAC), kernel breadth before cooking (KBBC), kernel breadth after cooking (KBAC), length/breadth ratio (L/B), elongation ratio (ER), alkali spreading value (ASV) and aroma was carried out as described in Basavaraj *et al.* (2010).

## Results

### Marker-assisted selection for BB and blast resistance gene(s)

Marker-assisted foreground selection for BB resistance genes *xa13* and *Xa21*, and blast resistance gene *Pi54* was carried out in the BC_1_F_1_ generation using gene-based/linked markers *xa13-prom*, pTA248 and RM206, respectively. Among the 188 BC_1_F_1_ plants analysed for the presence of the *Pi54* gene using RM206, 80 plants were heterozygous for the resistance allele. All 80 plants heterozygous for the *Pi54* gene-linked marker were then subjected to foreground selection for the genes *xa13* and *Xa21* using the respective gene-based markers, which led to the identification of 18 plants homozygous for the two BB resistance genes*.* The plant exhibiting maximum similarity to the recurrent parent was then backcrossed with the recurrent parent to generate 145 BC_2_F_1_ seeds. Based on foreground selection, five BC_2_F_1_ plants found to be heterozygous for *Pi54* and homozygous for *xa13*, *Xa21* as well as having desirable agro-morphological and grain quality characteristics were advanced to generate BC_2_F_2_ populations. Twenty-two plants homozygous for the three genes *xa13*, *Xa21* and *Pi54* were identified in BC_2_F_2_ populations and advanced to BC_2_F_5_ generation through pedigree breeding with selection for superior plant phenotype and grain and cooking quality characters of Basmati rice.

### Marker-assisted selection for the major QTL *qSBR11-1* for ShB resistance

A total of 22 BC_2_F_5_ families homozygous for all three genes were further evaluated for a major QTL for ShB resistance, *qSBR11-1*, using flanking markers RM224 and RM443. A total of seven families were found to amplify ‘Tetep’-specific alleles for both flanking markers, while one plant was found to be heterozygous for both marker alleles. These seven families possessing the major QTL *qSBR11-1* for ShB resistance were also re-confirmed for the presence of *Pi54*, *xa13* and *Xa21*, and advanced to BC_2_F_6_ generation.

### Marker-assisted background analysis

**Fig. 1:**
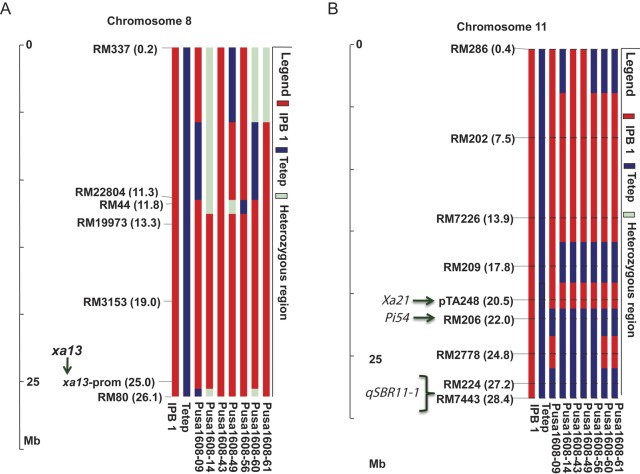
**Analysis of genome introgression associated with resistance genes/QTL.** (A) *xa13* on chromosome 8 and *Xa21, Pi54* and *qSBR11-1* on chromosome 11 (B) in ‘Pusa1608’ families.

Sixty of the 435 sequence tagged microsatellite site (STMS) markers were found to be polymorphic between the parental lines and were used for background analysis of seven selected BC_2_F_5_ families (Table 2). The background analysis of the improved lines indicated RPG recovery ranging from 76.25 % in ‘Pusa1608-06-16-3-60’ to 89.50 % in ‘Pusa1608-06-7-10-14’. To identify the recombinants with least donor segment introgression, 22 markers were identified from the flanking regions (∼5 Mb on either side) of the gene *xa13* (25.01 Mb) on chromosome 8. However, only one marker RM80, flanking the gene *xa13* at a distance of 1.1 Mb, was found to be polymorphic and this was used to identify the recombinant line with minimum donor parent segment (Fig. 1A). Similarly, 43 markers in the genomic region spanning across the genes *Xa21* (20.54 Mb), *Pi54* (21.97 Mb) and *qSBR11-1* (27.2-28.4 Mb) on chromosome 11 were analysed for polymorphism between the parental lines, of which only two markers, RM209 and RM2778, were found to be polymorphic (Fig. 1B). The advanced breeding line ‘Pusa1608-06-7-5-9’ had the least donor segment in the carrier chromosome 11 (<4.25 Mb donor segment in the genomic region of *Xa21* and *Pi54*; <1.8 Mb donor segment around ShB QTL *qSBR11-1*), while the rest of the lines showed higher donor parent genome introgression. The polymorphic marker RM2778, located between *Pi54* and *qSBR11-1*, also identified two other advanced breeding lines (‘Pusa1608-06-16-3-60’ and ‘Pusa1608-06-16-3-61’) with minimum donor segments around ShB QTL *qSBR11-1*, but they had a larger donor segment in the genomic region of *Xa21* and *Pi54* (<6.2 Mb) compared with ‘Pusa1608-06-7-5-9’.

**Table 2:** Agronomic performance of the improved lines of Pusa1608.

Designation	DFF (days)	PH (cm)	NT	PL (cm)	FG/P	SF (%)	TW (g)	Y/P (g)	RPG (%)
Pusa1608-06-7-5-9	107	93.20	15	28.60	148	90.58	24.16	21.10	78.33
Pusa1608-06-7-10-14	115	90.80	12	32.20	155	88.88	21.50	19.70	89.50
Pusa1608-06-13-1-43	103	89.60	10	27.00	121	86.88	21.95	17.80	89.17
Pusa1608-06-14-2-49	100	94.60	11	24.20	135	79.89	22.20	18.70	85.00
Pusa1608-06-15-4-56	108	95.20	12	27.80	155	89.86	24.20	21.20	85.00
Pusa1608-06-16-3-60	108	98.20	14	33.80	141	86.73	23.21	19.70	76.25
Pusa1608-06-16-4-61	110	91.40	14	30.20	140	88.25	23.61	21.10	80.83
Improved Pusa Basmati 1	105	93.40	13	32.20	146	89.50	22.79	19.20	–
CD (0.05)	2.63	0.44	2.78	3.01	7.91	1.43	0.31	3.12	–

DFF, days to 50% ﬂowering; PH, plant height; NT, no. of tillers; PL, panicle length; FG/P, filled grains/panicle; SF, spikelet fertility; TW, thousand grain weight; Y/P, yield per plant; RPG, per cent recurrent parent genome recovery.

### Agronomic performance

The agronomic performance of advanced breeding lines was evaluated during Kharif (autumn) 2010 (Table 2). There were no significant differences between the improved lines and the recurrent parent ‘Improved Pusa Basmati 1’ for important agronomic traits such as (i) NT and (ii) PL. Days to 50 % flowering of advanced lines ranged from 100 to 115 days with a mean of 107.8 days, which was similar to ‘Improved Pusa Basmati 1’ (105 days). The PL was as low as 24.20 cm in ‘Pusa1608-06-14-2-49’ to 33.8 cm in ‘Pusa1608-06-16-3-60’. This compares with 32.20 cm in ‘Improved Pusa Basmati 1’. Spikelet fertility and TW of some of the advance breeding lines were better than those of the recurrent parent. The Y/P in the advanced lines was on a par with the recipient parent ‘Improved Pusa Basmati 1’. All seven selections were characterized by semi-dwarf plant height, sturdy stems, and dark and green flag leaves. These are the prominent traits distinguishing the recurrent parent ‘Improved Pusa Basmati 1’ from the donor ‘Tetep’.

### Evaluation of grain and cooking quality

**Fig. 2:**
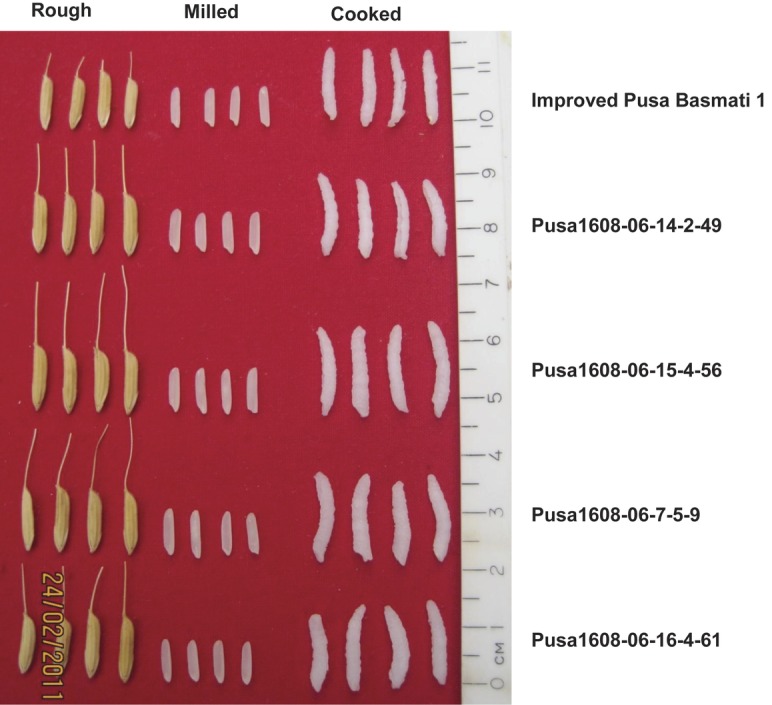
Rough, milled and cooked rice of ‘Improved Pusa Basmati 1’ and ‘Pusa1608’ lines.

The grain and cooking quality of the Pusa1608 lines was evaluated and is presented in Table 3. All the improved lines possessed extra long, slender grains similar to those of the recurrent parent ‘Improved Pusa Basmati 1’ (Fig. 2). The KLBC of the advanced breeding lines ranged from 7.80 mm in ‘Pusa1608-06-7-5-9’ to 8.73 mm in ‘Pusa1608-06-16-3-60’. The L/B ratio of the improved lines was in the range 4.03–5.46 as compared with 5.0 for ‘Improved Pusa Basmati 1’. One of the improved lines (‘Pusa1608-06-7-10-14’) showed a significantly superior elongation ratio (1.83) compared with the recurrent parent ‘Improved Pusa Basmati 1’. Although this line had significantly lower KLBC, it was able to make up the KLAC on a par with the recurrent parent because of its significantly superior elongation ratio. The alkali spreading value and aroma of all the advanced breeding lines were similar to those of ‘Improved Pusa Basmati 1’.

**Table 3:** Grain and cooking quality characteristics of the improved lines of Pusa1608.

Designation	Grain shape	KLBC (mm)	KBBC (mm)	L/B ratio	KLAC (mm)	KBAC (mm)	ER	ASV	Aroma
Pusa1608-06-7-5-9	Extra long	7.80	1.93	4.03	12.40	2.80	1.59	7	2
Pusa1608-06-7-10-14	Extra long	8.07	1.67	4.84	14.80	2.60	1.83	7	2
Pusa1608-06-13-1-43	Extra long	8.33	1.93	4.31	14.00	2.53	1.68	7	2
Pusa1608-06-14-2-49	Extra long	8.00	1.87	4.29	13.67	2.60	1.71	7	2
Pusa1608-06-15-4-56	Extra long	8.20	1.73	4.73	14.40	2.47	1.76	7	2
Pusa1608-06-16-3-60	Extra long	8.73	1.60	5.46	13.47	2.33	1.54	7	2
Pusa1608-06-16-4-61	Extra long	7.93	1.67	4.76	13.13	2.33	1.66	7	2
Improved Pusa Basmati 1	Extra long	8.33	1.67	5.00	14.73	2.53	1.77	7	2
Tetep	Medium	5.58	1.93	2.89	8.40	2.87	1.50	3	0
CD (0.05)	–	0.13	0.01	0.09	0.24	0.19	0.03	–	–

Grain shape, extra long: >7.50 mm, medium: 5.51–6.60 mm; iKLBC, kernel length before cooking; KBBC, kernel breadth before cooking; L/B ratio, length/breadth ratio; KLAC, kernel length after cooking; KBAC, kernel breadth after cooking; ER, elongation ratio; ASV, alkali spreading value (1–2 high and 6–7 low); aroma, 0 absent and 2 strong.

### Disease reaction for BB, blast and ShB

The advanced lines in the background of ‘Improved Pusa Basmati 1’ were screened for resistance to BB, blast and ShB with ‘Improved Pusa Basmati 1’ and ‘Tetep’ as checks using standard procedures (Table 4). The donor parent ‘Tetep’ was found to be resistant to the most virulent BB isolate ‘Kaul’ (lesion length 2.20 cm), all four isolates of blast under artificial inoculation (score of 0 for all four isolates) and in UBN (score of 0) when tested in the UBN of the Agricultural Research Station at Mugad for leaf and neck blast. It was also resistant to the highly virulent ‘Kapurthala’ isolate (*Rs-K*) of ShB. In contrast, the recurrent parent ‘Improved Pusa Basmati 1’ was resistant to BB (lesion length of 1.80 cm) and highly susceptible to both blast (both under artificial conditions—a score of 5 for all the isolates and in the UBN a score of 7 for leaf and neck blast) and ShB (score of 7).

**Table 4:** Reaction of the improved lines of Pusa1608 to BB, blast and ShB.

Details	BB	Blast						Sheath blight
	Disease reaction (lesion length)	Disease reaction under artificial inoculation (0–5 scale)				Disease reaction in UBN trials (0–9 scale) ARS, Mugad		Disease reaction (0–9 scale)
	Kaul isolate	Mo-ni-007	Mo-ni-012	Mo-ni-018	Mo-ni-019	Leaf blast	Neck blast	*Rs-K* isolate
Pusa1608-06-7-5-9	3.56	1	2	0	0	2	3	3
Pusa1608-06-7-10-14	2.88	2	1	2	0	3	3	3
Pusa1608-06-13-1-43	2.50	0	0	1	3	2	7	3
Pusa1608-06-14-2-49	2.25	1	0	0	1	2	5	3
Pusa1608-06-15-4-56	1.88	1	2	1	0	2	3	3
Pusa1608-06-16-3-60	2.33	2	3	0	1	4	5	3
Pusa1608-06-16-4-61	1.40	1	2	0	1	2	3	3
Improved Pusa Basmati 1	1.80	5	5	5	5	9	9	7
Tetep	2.20	0	0	0	0	0	0	1

UBN, Uniform Blast Nursery; DAI, days after inoculation; RLH, relative lesion height.

Resistance to BB was evaluated on the basis of lesion length. A lesion length <6 cm was considered as resistant, 6–10 cm as moderately resistant, 10–15 cm as moderately susceptible and >15cm as susceptible. For evaluation of blast resistance under artificial inoculation a 0–5 scale was adopted, where scores of 0–2 were considered as resistant, 3 as moderately resistant and 4–5 as susceptible, while in UBN a 0–9 scale was used, wherein scores of 0–3 were considered as resistant, 4–5 as moderately resistant, 6 as moderately susceptible and 7–9 as susceptible. For evaluation of ShB resistance, a 0–9 scale based on RLH was used, where 0 was considered as highly resistant (RLH = 0), 1 as resistant (RLH < 20), 3 as moderately resistant (RLH: 20–30), 5 as moderately susceptible (RLH: 31–45), 7 as susceptible (RLH: 45–65) and 9 (>65) as highly susceptible.

**Fig. 3:**
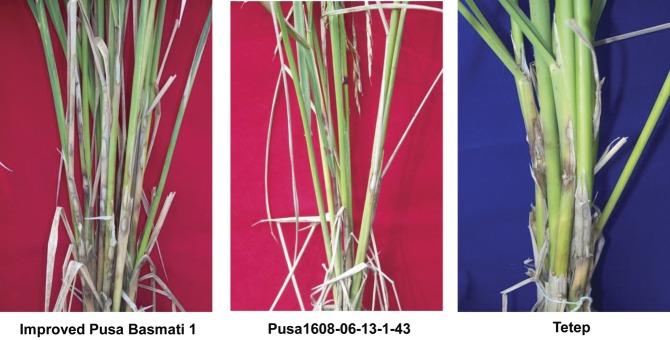
Sheath blight symptoms 25 days after inoculation in the parents and an improved line.

All seven improved lines were resistant to BB and the lesion lengths ranged from 1.40 cm in ‘Pusa1608-06-16-4-61’ to 3.56 cm in ‘Pusa1608-06-7-5-9’, in comparison with 1.80 and 2.20 cm in ‘Improved Pusa Basmati 1’ and ‘Tetep’, respectively. Evaluation of all these advanced lines for blast resistance showed a differential reaction pattern to different isolates, with five out of seven entries showing resistant reactions and two showing moderately resistant reactions with different isolates. Four out of seven advanced breeding lines (‘Pusa1608-06-7-5-9’, ‘Pusa1608-06-7-10-14’, ‘Pusa 1608-06-15-4-56’ and ‘Pusa1608-06-16-4-61’) were found to be resistant to blast both under artificial inoculation and under natural epiphytotic conditions in a hotspot location. All the advanced lines (‘Pusa1608’ lines) were moderately resistant (score of 3) under artificial screening with the virulent isolate of ShB (Fig. 3) when compared with the susceptible reaction shown by ‘Improved Pusa Basmati 1’ (score of 7).

## Discussion

Basmati rice production is often constrained by several biotic stresses, among which diseases such as BB, blast, and ShB impose both severe yield and quality losses. The development of improved ‘Pusa Basmati 1’ with BB resistance through MAS of two BB resistance genes *xa13* and *Xa21* (Gopalakrishnan *et al*. 2008) helped address the problem of BB. However, the severe incidence of blast disease, especially neck blast, is a major constraint in Basmati rice production. Therefore, the present study was undertaken with the objective of transferring a gene, *Pi54*, providing broad-spectrum resistance to blast disease using the cultivar ‘Tetep’ as the multiple disease-resistant donor with resistance to blast, BB and ShB.

Marker-assisted selection offers a simpler and more efficient and accurate way to breed improved cultivars. It is especially helpful for breeding disease resistance compared with selection-only based on phenotype screening. Marker-assisted pyramiding of major genes/QTLs has helped in the past to reduce susceptibility to major diseases such as BB (Huang *et al.* 1997; Singh *et al.* 2001; Joseph *et al.* 2004; Zhang *et al.* 2006; Gopalakrishnan *et al.* 2008; Sundaram *et al.* 2008, 2009; Basavaraj *et al.* 2009, 2010), blast (Hittalmani *et al.* 2000; Zhou *et al.* 2011) and ShB (Wang *et al.* 2011). However, there are no reports of the transfer of genes/QTLs conferring resistance to multiple diseases in rice. Despite many advances made in rice genomics and breeding, we are only aware of two reports that combined resistance to multiple diseases in rice using a transgenic approach (Datta *et al*. 2002; Narayanan *et al*. 2002), and there is no previous report on the development of multiple disease-resistant rice cultivars through MAS.

With no pre-existing sources of resistance against blast, BB and ShB in the Basmati rice germplasm, there was a need to transfer the genes from non-Basmati sources. One of the most important limitations in transferring disease resistance from other sources is the impairment of grain and cooking quality traits, which are unique to Basmati rice. Therefore, we adopted an MABB strategy involving marker-assisted foreground selection for gene(s)/QTLs of interest along with stringent phenotypic selection for grain and cooking quality traits (Singh *et al*. 2011).

The disease reaction for BB resistance in some of the advanced breeding lines showed greater resistance than the recurrent parent ‘Improved Pusa Basmati 1’. This could be due to complementarity of genes from the donor parent ‘Tetep’, which have been reported to carry other BB-resistant genes, namely *Xa1, Xa2, Xa12* and *Xa16* (Nelson *et al*. 1994; Goel *et al.* 1998). However, the markers linked to the *Xa2* gene (He *et al.* 2006) were found to be monomorphic and the presence of these genes could not be ascertained in the improved lines. However, because ‘Tetep’ is a highly valuable source of genes for resistance to multiple diseases, the contribution from this donor parent cannot be ruled out.

The differential response of the improved lines for blast resistance with different isolates and in the hotspot location could be attributed to the differences in the genetic make-up of the improved lines (Singh *et al.* 2012). Although screening of the backcross-derived lines under artificial inoculation is time consuming and laborious, it does offer scope for selecting genotypes with genetic backgrounds offering higher levels of resistance to disease in the presence of one major gene such as *Pi54*. Therefore, meticulous screening of the advanced/elite lines for traits of interest (such as disease resistance in this case) in the advanced stages of the breeding programmes can help in identifying genotypes with superior levels of trait expression. All the improved lines were moderately resistant to ShB compared with the donor parent ‘Tetep’, which exhibited a higher level of resistance (score 1). The complete resistance of ‘Tetep’ can be attributed to the presence of 12 different QTLs spanning the genome (Channamallikarjuna *et al*. 2010). Although MAS for pyramiding multiple minor resistant QTLs is a possible contributor to achieving a higher level of resistance in the advanced lines, it risks being accompanied by the possibility of bringing undesirable traits into the advanced breeding lines due to linkage drag (Kou and Wang 2012).

Marker-assisted background selection in early backcross generations has been advocated for quick recovery of the RPG (Chen *et al*. 2001; Joseph *et al*. 2004). Parallel to this study, Channamallikarjuna *et al*. (2010) independently reported markers linked to as many as 12 QTLs for ShB resistance in Tetep, the donor for the blast resistance gene *Pi54*, which included one major QTL, *qSBR11-1*, for ShB resistance in the genomic region of *Pi54* in chromosome 11. Therefore, we used the flanking markers RM224 and RM7228 linked to *qSBR11-1* to successfully identify the superior breeding lines integrating the blast resistance gene *Pi54* also at BC_2_F_5_ stage. A strict adoption of marker-assisted background selection in the carrier chromosome for precise transfer of the blast resistance gene *Pi54* would have meant the removal of possible linkage drag associated with *Pi54* in chromosome 11. This would have eliminated the ShB resistance QTL *qSBR11-1* from the advanced breeding lines. However, the delayed application of marker-assisted background selection has been favourable in retaining the recombinants possessing the ShB resistance QTL *qSBR11-1* without compromising either agronomic performance or Basmati-type grain and cooking quality characteristics. This has been made possible by combining stringent phenotypic selection with a major focus on agronomic and quality traits for selection of the plants and MAS for gene(s) of interest (Gopalakrishnan *et al.* 2008). Background analysis of the advanced lines using 60 polymorphic STMS markers across the genome revealed that up to 89.50 % of the RPG had been recovered in only two backcross generations. Among these markers, four have been reported to be linked with important grain and cooking quality traits, *viz.* RM276 for gelatinization temperature and ASV (Tian *et al*. 2005; Wang *et al*. 2007), RM80 for aroma (Amarawathi *et al*. 2008), RM202 associated with KL (Agrama *et al*. 2007) and RM247 linked to the QTL, *qRLW12*, determining L/B ratio (Li *et al*. 2010) (data not presented). It was observed that at all these marker loci, all seven advanced elite genotypes had recovered the recurrent parent-specific alleles, possibly due to stringent phenotypic selection for grain and cooking quality characters. Although four of the improved lines had KBBC significantly different from ‘Improved Pusa Basmati 1’, the KBBC values of all the lines were ≤1.93 mm. As per the notified standards for Basmati rice, the KBBC should be <2 mm; therefore, slightly thicker grains but within the standard norm is a desirable attribute, as this would help in reducing the percentage of broken grains and increasing the head rice recovery. The elite genotype ‘Pusa1608-06-7-10-14’ was found to be resistant to BB and blast, moderately resistant to ShB, while possessing better grain and cooking quality with a yield on a par with the recurrent parent ‘Improved Pusa Basmati 1’.

Although marker-assisted background selection is very useful in rapid reconstitution of the RPG, it can be very demanding in terms of application in a breeding programme due to several constraints, including identifying useful polymorphisms, its cost and timely execution (Singh *et al*. 2012). Therefore, a more pragmatic approach involving marker-assisted foreground selection for trait(s) of interest, in combination with phenotypic selection for specific characters unique to the recurrent parent, would help not only in transferring the trait of interest into the recurrent parent but also in developing superior lines with additional desirable traits from the donor parent. This would be particularly relevant when the donor parent has many desirable traits in addition to the target trait being transferred, e.g. ShB resistance QTL *qSBR11-1* in addition to *Pi54* for blast resistance from ‘Tetep’. Altogether, MAS has been effective in combining three genes (*xa13*, *Xa21*, *Pi54*) and one QTL (*qSBR11-1*) conferring resistance to BB, blast and ShB diseases in an elite Basmati rice cultivar, ‘Improved Pusa Basmati 1’.

## Conclusions and forward look

This is the first report of marker-assisted transfer of genes conferring resistance to three different diseases in rice. The genes involved are *xa13* and *Xa21* for BB resistance; *Pi54* for blast resistance; and a major QTL *qSBR11-1* for ShB resistance. These have been combined through MABB to lead to the development of improved lines that are highly resistant to BB and blast, and moderately resistant to ShB. The improved lines have desirable Basmati grain and cooking quality characteristics, in tandem with inbuilt resistance to BB, blast and ShB, and yield on a par with ‘Improved Pusa Basmati 1’. These multiple biotic stress-resistant lines will now be evaluated under multilocation trials for release to farmers as improved Basmati cultivars. They will also be a unique source for BB, blast and ShB resistance genes in future Basmati breeding programmes.

## Sources of funding

The research was funded by the Indian Council of Agriculture Research, New Delhi, India, under the ICAR-Network Project on Gene Pyramiding (2005–2008).

## Contributions by the authors

All the authors contributed to a similar extent overall.

## Conflicts of interest statement

None declared.
